# Epidemiologic features and potential year of life lost of scrub typhus in China: A nationwide surveillance analysis (2006– 2023)

**DOI:** 10.1371/journal.pntd.0013666

**Published:** 2025-10-29

**Authors:** Pei-Ying Peng, Lei Xu, Ji-Qin Sun, Ting-Liang Yan, Zi-Liang Li, Hui-Ying Duan, Li-Juan Ma, Ya Zu

**Affiliations:** 1 Institute of Microbiology of Qujing Medical College, Qujing, Yunnan Province, China; 2 Department of Clinical Laboratory, Qujing Second People’s Hospital, Qujing, Yunnan Province, China; University of Liverpool, UNITED KINGDOM OF GREAT BRITAIN AND NORTHERN IRELAND

## Abstract

**Background:**

Scrub typhus, an overlooked vector-borne disease in mainland China, has shown shifting epidemiological patterns in recent decades, yet comprehensive assessments of its spatiotemporal trends and disease burden—including premature mortality quantified by years of potential life lost (YPLL)—remain limited. This study aimed to characterize the epidemiological trends, spatiotemporal patterns, and disease burden of scrub typhus in mainland China, with a focus on estimating YPLL.

**Methods:**

Nationwide scrub typhus case data were extracted from the China Information System for Disease Control and Prevention (CISDCP). Time-series analysis, spatial autocorrelation analysis, and spatiotemporal clustering analysis (SaTScan) were performed, and years of potential life lost (YPLL) were calculated to explore the epidemiological characteristics and spatiotemporal patterns of the scrub typhus in China. Negative binomial regression analysis was used to explore the association between scrub typhus and environmental variables.

**Results:**

There were 283273 cases and 103 deaths reported. 2006–2023, the average yearly incidence was 1.14 cases per 100,000 people. From 0.10 per 100,000 population in 2006 to 2.37 per 100,000 population in 2023, the annual incidence rose dramatically. In 2023, there were 1,150 impacted counties, up from 226 in 2006. Yunnan (84795), Guangdong (70 013), Guangxi (30147), Anhui (20492) and Jiangsu (16760) were the top five provinces in terms of reported cases, accounting for 78.44% of all scrub typhus cases. The disease, which was endemic in southern China from 2006 to 2009, has spread to every province, particularly in northernmost and western of China. October has the highest seasonal index (2.53), followed by July and August. The majority of affected groups were women (52.90%), farmers (76.11%), and those between the ages of 40 and 59 (39.98%). The percentage of cases involving those 60 and older rose from 22.83% in 2006 to 37.90% in 2023. Spatial autocorrelation analyses showed a significant positive spatial correlation for scrub typhus incidence in all years except 2006–2011, showing a clustering distribution. The LISA cluster maps showed “high-high” clusters expanding in southern China, and “low-low” clusters were growing in northern areas. The results of negative binomial regression model revealed significant positive effects of temperature with a 1-month lag (IRR = 1.17, p < 0.001), rainfall with a 2-month lag (IRR = 1.008, p = 0.002), NDVI (IRR = 1.07, p = 0.008), and incidence in neighboring provinces (IRR = 1.05, p = 0.013) on scrub typhus risk. YPLL analysis highlighted substantial mortality impacts, particularly the age groups among males that most contributed to the losses were 40–49 years and 0–4 years (28.32% and 27.99%, respectively), while the highest frequencies of YPLL among females were observed between 50 and 59 years old (40.97%) and 60–69 years old (21.27%).

**Conclusions:**

Based on results, we recommend prioritizing surveillance and resource allocation to high-risk areas including Guangdong, Yunnan, Guangxi, and Fujian provinces, as well as emerging northern regions (e.g., Anhui, Shandong) exhibiting rapid geographic expansion. Health interventions should target farmers (particularly females) and adults aged ≥60 years in rural areas. Meanwhile, efforts should accelerate vaccine development for high-risk occupational groups. Vector control and prevention campaigns should be intensified during critical pre-peak windows: the outbreak peaked in October.

## Author summary

In China, scrub typhus, a disease caused by *Orientia tsutsugamushi* and spread by its vector, is still a major public health issue. Scrub typhus’s epidemiological patterns, spatiotemporal distribution, and health effects in mainland China from 2006 to 2023 were analyzed in this study. Seasonal clustering (June- November, 88.45% of cases) peaked in October (seasonal index = 2.53), and annual incidence rates rose dramatically from 0.10 to 2.37 per 100,000 population, with a total of 283,273 confirmed cases and 103 deaths included. Geographically, cases were mainly concentrated in southern provinces (Yunnan, Guangdong, Guangxi, Anhui, Jiangsu), accounting for 78.44% of totals, and expanded northward over time, with 29 provinces reporting cases by 2023. Females (1.12:1 ratio) and individuals aged 40–59 years (39.98%) were most affected, while farmers represented 76.11% of cases. Case-fatality ratios fluctuated, peaking at 0.27% in 2010, and mortality disproportionately affected males aged 40–49 years and females aged 50–59 years based on years of potential life lost (YPLL) calculations. These findings highlight the need for targeted prevention strategies, enhanced surveillance, and vector control measures to address rising incidence and northward expansion, particularly in high-risk populations and newly endemic regions.

## Introduction

In Asia-Pacific, including China, scrub typhus, a deadly zoonosis spread by chigger mites, reemerged as a significant neglected tropical disease [1,2]. Scrub typhus has historically been endemic in the southern regions of China, where it was first observed as far back as 313 A.D. in antiquity. However, its distribution has spread to northern regions and its prevalence has increased in recent decades due to a number of variables, including increased human activity and climate change [3–5]. Mites are the only vector and host of *Orientia tsutsugamushi*. Rodents, although acting as dead-end hosts like humans, serve as a major source of nutrition and major host for chigger mites. Scrub typhus can cause life-threatening complications such as abrupt hearing loss and multiple organ failure, as well as present as undetectable or unusual feverish conditions [6,7]. Scrub typhus is an emerging and re-emerging illness that has been neglected and frequently misdiagnosed due to its undifferentiated clinical symptoms (e.g., headache, myalgia, nausea), which can be confused with many other diseases. Therefore, early detection and treatment can greatly lower the complication and fatality rate, and there are currently no long-lasting, broadly-protective vaccinations against scrub typhus [8].

The incidence and prevalence of scrub typhus in China have shown a marked upward trajectory. Nationwide surveillance from 2006 to 2014 recorded 54558 cases and 37 deaths, with the average annual incidence rising sharply from 0.10 to 0.46 per 100,000 population [9]. This trend is corroborated by regional studies; for example, Yunnan Province reported 27,838 cases and 11 deaths from 2006 to 2017, while Guangdong Province experienced persistent high incidence, with hotspots like Shenzhen documenting an average annual rate of 0.165 per 100,000 from 2006 to 2013, peaking at 0.43 per 100,000 in 2013 [10,11]. The overall mortality remains relatively low (approximately 0.04% of cases) but is elevated in vulnerable populations such as children and the elderly, with case fatality rates reaching up to 10% in pediatric groups due to delayed diagnosis and treatment [12]. This increase in incidence is partly attributed to underreporting and diagnostic challenges, as scrub typhus is often clinically misidentified due to nonspecific symptoms like fever and eschar formation, leading to insufficient public awareness and delayed interventions [13].

Predominant risk factors for scrub typhus included agricultural work, specific vegetation ex-posure, other outdoor activities, risky personal health habits, and exposure to rodents, livestock, or poultry [14]. farmers, outdoor laborers, and individuals in rural or peri-urban settings face higher exposure risks, with occupational activities such as farming, forestry, and construction facilitating contact with mite-infested habitats [13]. Furthermore, socioeconomic determinants are one of the risk factors for scrub typhus. Socioeconomic factors, including population mobility, land-use changes (e.g., afforestation projects), and limited access to healthcare in impoverished regions, further amplify transmission. Studies in Taiwan and Fujian have linked outbreaks to environmental degradation and climatic anomalies, such as increased temperatures and extreme weather events, which alter vector ecology and expand endemic foci [15].

Despite its growing burden, comprehensive assessments of scrub typhus in China remain limited, particularly regarding premature mortality. There are significant gaps in our knowledge of the disease burden of scrub typhus and nationwide spatiotemporal patterns since most existing research focuses on descriptive epidemiology or localized spatiotemporal clusters, neglecting integrated metrics like years of potential life lost (YPLL) that quantify societal impact [9,10,16]. And, years of potential life lost (YPLL), has not been systematically evaluated for scrub typhus in China. This study aims to address these gaps by analyzing long-term (18 years) of national surveillance data (2006–2023) to describe the temporal trends in scrub typhus incidence, detect spatiotemporal clusters of scrub typhus cases at the provincial level, identify the physical environmental variables associated with scrub typhus incidence, and assess YPLL across demographic groups. Knowledge of the geographical distribution and burden of scrub typhus is essential for determining the allocation of limited resources necessary for scrub typhus control. The study results will help health administration officers and public health workers implement effective intervention measures and optimal resources allocation aimed at high-risk areas and populations.

## Materials and methods

### Ethics statement

In the current study, data on scrub typhus cases were gathered from online sources, with no human or animal samples included. As a result, the research does not require ethical approval or participant consent. Meanwhile, all data were kept anonymous, and we all agreed that all techniques were carried out in compliance with applicable standards and regulations.

### Data collection and management

Scrub typhus is a vector-borne disease that needs to be reported in China. China’s national scrub typhus surveillance program evolved over three periods. Initially (1952–1989), mail-based monthly aggregated data reporting was used. Voluntary reporting started in some areas in 1952, and it became a statutorily notifiable disease in 1955, requiring reporting of all suspected, probable, and laboratory-confirmed cases. Reporting paused from 1990-2005 after scrub typhus was removed from notifiable diseases in 1990 due to its low threat, leaving no nationwide data during this period. From 2006 to 2016, internet-based individual case reporting was adopted when it was added to China CDC’s National Notifiable Infectious Disease Reporting System in 2006 [17]. The law requires attending physicians to use the China Information System for Disease Control and Prevention (CISDCP) to report the disease to the China Center for Disease Control and Prevention. Basic demographic and clinical information, such as gender, age, occupation, residential address, date of symptom start, laboratory diagnosis, and clinical outcome, are included in scrub typhus case reports. Data of 31 provinces in mainland China from January 2006 through December 2023 were obtained from CISDCP. According to the diagnostic standards published by the People’s Republic of China’s Ministry of Health, every case of scrub typhus was verified. In addition to clinical symptoms such as high fever, and physical signs including lymphadenopathy, skin rash, eschar, or ulcers, epidemiological exposure histories (visiting an endemic area and coming into contact with chiggers or rodents within three weeks prior to the onset of illness), and at least one laboratory diagnosis—a 4-fold or greater increase in serum IgG antibody titers between acute and convalescent sera using the indirect immunofluorescence antibody assay (IFA), the detection of *O. tsutsugamushi* in clinical specimens using polymerase chain reaction (PCR), or isolation of *O. tsutsugamushi* from clinical specimens—are required for a confirmed case of scrub typhus [18,19]. A summary of the case definitions is available in S1 Text. Crucially, for the analyses presented in this article, we exclusively utilized cases that met the stringent criteria for a laboratory-confirmed diagnosis of scrub typhus. Monthly meteorological data, namely average temperature and rainfall, for the same period were collected from the China Meteorological Data Sharing Service System (https://data.cma.cn/data/). The average data of vegetation normalization index (NDVI) were collected from the MOD13A3 (10.5067/MODIS/MOD13A3.006).

### Statistical analysis

By dividing the number of human scrub typhus cases by the equivalent population at the end of a particular year, we were able to determine the yearly incidence rate. The National Bureau of Statistics of China provided us with the population data that we utilized to determine incidence rates [20]. By dividing the number of scrub typhus cases by the number of human deaths, we were able to get the case-fatality ratio. Seasonal indices were calculated as the ratio of the monthly mean incidence to the overall mean incidence during the study period. A seasonal index greater than 1 indicates a higher than average incidence for that month, while a value less than 1 indicates a lower than average incidence [21]. IBM SPSS Statistics version 24.0 was used to perform time-series analysis for scrub typhus cases (IBM Corp., NY, USA).

### Spatial autocorrelation analysis

We used GeoDa and ArcGIS software to conduct both global and local spatial autocorrelation analyses. Moran’s *I* was used to detect the existence of global spatial autocorrelation in scrub typhus incidence in China and to measure their correlational strength. A Z-test was used to assess the statistical significance of Moran’s *I*. Moran’s *I* generally ranges from −1 to +1. If Moran’s *I* = 0, it means that there is no spatial autocorrelation and that the incidence of scrub typhus are distributed randomly throughout China. Positive spatial autocorrelation is shown by a value of Moran’s *I* > 0, with values nearer 1 suggesting a stronger autocorrelation. Moran’s *I* < 0 denotes a negative spatial autocorrelation, whereas values closer to -1 denote a higher degree of spatial variability [22]. The local indicators of the spatial autocorrelation map were used to assess the local cluster of the incidence of scrub typhus in China. The Local Indicators of Spatial Association (LISA) cluster map displays four types of clusters: high-high (H-H) clusters are places with high incidence surrounded by additional high incidence areas, low-low (L-L) clusters are places with low incidence surrounded by other low incidence areas, low-high (L-H) clusters are locations with low incidence surrounded by high incidence areas, and high-low (H-L) clusters are places with a high incidence that are surrounded by areas with a low incidence [23].

### Spatiotemporal clustering analysis

High risk clusters of scrub typhus were detected with a retrospective space-time scan statistic based on a discrete Poisson model, using SaTScan software (version 10.1.2). The space-time scan statistic is defined by a cylindrical window with a circular geographical base which is centered on the centroids of areas, and with height corresponding to time [24]. The null hypothesis assumed that the relative risk (RR) of the incidence was the same within the window as compared with outside [25]. The base and the height of the windows are dynamic in order to detect possible sub-clusters. The difference of the incidence inside and outside the windows was evaluated by the Log Likelihood Ratio (LLR):


$$LLR=log\{{\left(c/n\right)}^{c}{\left[(C-c)/(C-n)\right]}^{\left(C-c\right)}\}$$

Where *C* denotes the total number of cases; *c* is the number of observed cases inside the window; *n* is the number of expected cases inside the window. The window with largest LLR value is defined as the primary cluster; other windows with statistically significant LLR values are defined as secondary clusters. Statistical significance was evaluated in a Monte Carlo simulation method, and a window with a *P* value less than 0.05 was identified as a statistically significant cluster [26].

In this study, we performed the space-time scan statistic annually to observe the cluster changes and adjust for the temporal trend during the study period [27]. The maximum radius of circular base was set at 50% of the total population at risk and the maximum height of the cylinder was set at 50% of the total study period. The number of Monte Carlo replications was set to 999 and the significance level was set at 0.05.

### Association between scrub typhus and the environmental factors

To examine the association between monthly scrub typhus incidence and potential environmental factors at the provincial level, we aggregated the monthly scrub typhus incidence and potential environmental factors into a panel dataset and then conducted panel negative binomial regression analyses. The negative binomial distribution is suitable for the analysis of overly dispersed data [28]. Through verification, we found that our data of scrub typhus was over-dispersed. Negative binomial regression was selected to model over-dispersed count data. Analyses used SPSS Generalized Linear Models (GLM) with log-link function, specifying ‘Negative Binomial’ distribution and scale parameter = 1. The base model structure was:

ln (E[Cases]) = β₀ + β₁X₁ +... + βₖXₖ + ln (Population) + ε

where E[Cases] represents the expected mean count of cases. ln (Population) was an offset term to control for population size. Spatial adjacency was operationalized as the mean incidence of neighboring provinces. (calculated via SPSS ‘Aggregate’ function)

Pearson’s correlation analysis was used to evaluate the correlation between co-variables, and highly correlated variables with a threshold of Pearson correlation |r| > 0.7 were not entered in the model simultaneously [15,29].

The negative-binomial dispersion parameter (α) was estimated as 0.67 (95% CI: 0.59- 0.76), confirming that the data were over-dispersed and that the negative-binomial model is more appropriate than a Poisson model. Model fit was assessed using the log-likelihood, Akaike’s Information Criterion (AIC), and Bayesian Information Criterion (BIC), with values of 7842.31, 7924.55, and 8105.72 respectively. Residual diagnostics revealed that deviance residuals had a mean of

-0.03 and a variance of 1.02, the Q-Q plot showed no systematic deviation from the theoretical distribution, and Pearson residuals ranged from -3.1 to 3.4 with < 2% of observations beyond ±2, indicating adequate model fit.

### Estimates of years of potential life lost (YPLL)

We estimate YPLL based on the method proposed by Romeder and McWhinnie [30]. In this study, deaths between the ages of 1 and 89 were considered, and the age limit of 90 years was adopted, considering it to be more inclusive and closer to the estimated life expectancy. Life expectancy at each age was taken from the life tables of the corresponding year; for example, the 2020 national averages (75.98 for males and 80.88 for females). YPLL and indicators derived from it were stratified by sex, age group.

We calculated the YPLL using the following formula, where *e* represents the life expectancy in years. *i* denotes the age group, typically calculated as the midpoint of the age group. *a*_*i*_ represents the remaining age, calculated as *a*_*i *_= *e*-(*i* + 0.5), which signifies the remaining years of life until reaching the age when death occurs at a certain age (group). *d*_*i*_ represents the number of deaths in a specific age group.


$$YPLL=\sum_{i=0}^{e}a_{i}d_{i}$$

## Results

### Descriptive epidemiology

**Fig 1:**
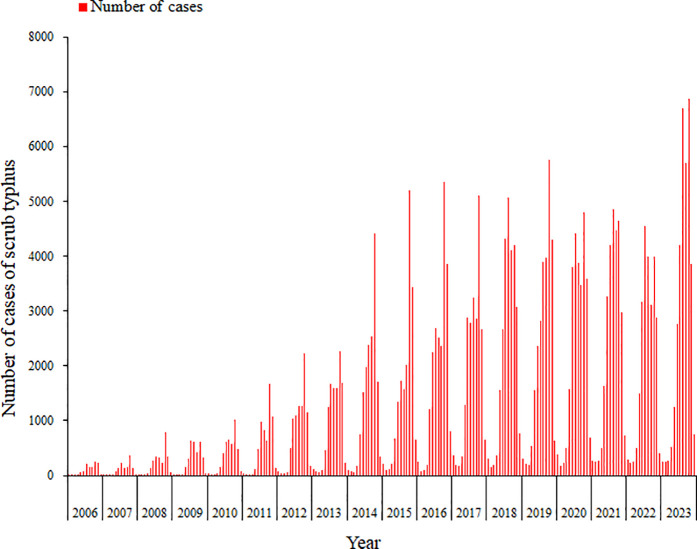
Time distribution of scrub typhus cases in China, 2006 − 2023.

From 2006 to 2023, a total of 283,273 scrub typhus cases and 103 deaths were reported nationwide in China. Over this 18-year period, the average annual incidence rate was 1.14 per 100,000 population. Both the number of reported cases and the incidence rate demonstrated significant upward trends, with the incidence rate rising from 0.10 per 100,000 population in 2006 to 2.37 per 100,000 population in 2023. The mortality rate exhibited fluctuating trends over time, peaking at 0.0009 per 100,000 population in 2016. The case fatality rate (CFR) reached its highest level of 0.27% in 2010, followed by low-amplitude fluctuations in subsequent years (see Table 1 and Fig 1). The female-to-male ratio of scrub typhus cases was 1.12:1, with a higher proportion of female patients. In terms of age distribution, the 0–19 years age group accounted for 11.15% of cases, the 20–39 years group for 13.43%, the 40–59 years group for 39.98%, and the ≥ 60 years group for 35.45%, indicating that scrub typhus cases were predominantly concentrated in the 40–59 years age group. From 2006 to 2023, the proportion of cases aged ≥60 years increased from 22.83% in 2006 to 37.90% in 2023, peaking at 39.64% in 2019. Farmers constituted the largest occupational group, representing 76.11% of all cases, followed by scattered children (5.30%), homemakers or unemployed individuals (5.09%), and students (4.44%) (Table 1)

**Table 1:** Epidemiological Characteristics of reported scrub typhus cases in China, 2006–2023.

Parameters	2006	2007	2008	2009	2010	2011	2012	2013	2014	2015	2016	2017	2018	2019	2020	2021	2022	2023
No. cases	1244	1332	2592	3235	4085	6020	8921	11104	16032	17280	21691	22556	26757	26519	27549	28086	24870	33400
IR	0.10	0.10	0.20	0.24	0.31	0.45	0.66	0.82	1.18	1.27	1.58	1.63	1.93	1.90	1.96	1.99	1.76	2.37
No. deaths	0	1	3	0	11	6	9	4	8	3	12	6	6	6	5	9	6	8
MR	0.0000	0.0001	0.0002	0.0000	0.0008	0.0004	0.0007	0.0003	0.0006	0.0002	0.0009	0.0004	0.0004	0.0004	0.0004	0.0006	0.0004	0.0006
CFR (%)	0.00	0.08	0.12	0.00	0.27	0.10	0.10	0.04	0.05	0.02	0.06	0.03	0.02	0.02	0.02	0.03	0.02	0.02
**Gender** Female	609 (49)	627 (47)	1222 (47)	1576 (49)	2046 (50)	3136 (52)	4698 (53)	6197 (56)	8811 (55)	9268 (54)	11756 (54)	12103 (54)	14319 (54)	14154 (53)	14760 (54)	15447 (55)	12932 (52)	17702 (53)
Male	635 (51)	705 (53)	1370 (53)	1659 (51)	2039 (50)	2884 (48)	4223 (47)	4907 (44)	7221 (45)	8012 (46)	9935 (46)	10453 (46)	12438 (46)	12365 (47)	12789 (46)	12639 (45)	11938 (48)	15698 (47)
**Age group** 0-19	263 (21)	261 (20)	432 (17)	702 (22)	788 (19)	924 (15)	1271 (14)	1434 (13)	1830 (11)	1608 (9)	2099 (10)	2251 (10)	2801 (10)	2264 (9)	2607 (9)	562 (2)	497 (2)	668 (2)
20-39	220 (18)	262 (20)	478 (18)	624 (19)	771 (19)	955 (16)	1287 (14)	1599 (14)	2269 (14)	2242 (13)	2915 (13)	3082 (14)	3700 (14)	3112 (12)	3384 (12)	2809 (10)	2238 (9)	2672 (8)
40-59	477 (38)	482 (36)	1023 (39)	1233 (38)	1570 (38)	2368 (39)	3648 (41)	4535 (41)	6496 (41)	7102 (41)	8750 (40)	9118 (40)	10735 (40)	10630 (40)	11104 (40)	14043 (50)	12684 (51)	17368 (52)
≥60	284 (23)	327 (25)	659 (25)	676 (21)	956 (23)	1773 (29)	2715 (30)	3536 (32)	5437 (34)	6328 (37)	7927 (37)	8105 (36)	9521 (36)	10513 (40)	10454 (38)	10672 (38)	9451 (38)	12692 (38)
**Occupation #** Farmers	728 (58.52)	855 (64.19)	1687 (65.08)	2078 (64.23)	2719 (66.56)	4249 (70.58)	6173 (69.20)	7847 (70.67)	11840 (73.85)	13224 (76.53)	16772 (77.32)	17546 (77.79)	18858 (70.48)	19209 (72.43)	21541 (78.19)	20578 (73.27)	19856 (79.84)	29840 (89.34)
Scattered children	90	98	198	312	394	478	705	788	939	839	1078	1123	1937	1954	1997	2865	2041	1032
Housework & unemployment	45	38	98	117	122	204	441	639	922	888	1081	1111	1886	1843	1385	1876	1154	964
Students	136	120	162	300	289	320	448	521	678	563	785	849	1543	1587	1056	1432	987	853
Retiree	46	34	67	45	81	125	197	275	326	427	449	431	869	543	475	765	364	389
Works	55	48	117	101	114	125	176	247	317	305	368	359	664	387	254	269	247	211

IR = Incidence rate (cases per 100,000 population); MR = Mortality rate (cases per 100,000 population); CFR = Case Fatality Rate; # columns do not add up to equal the total because of missing data.

**Fig 2:**
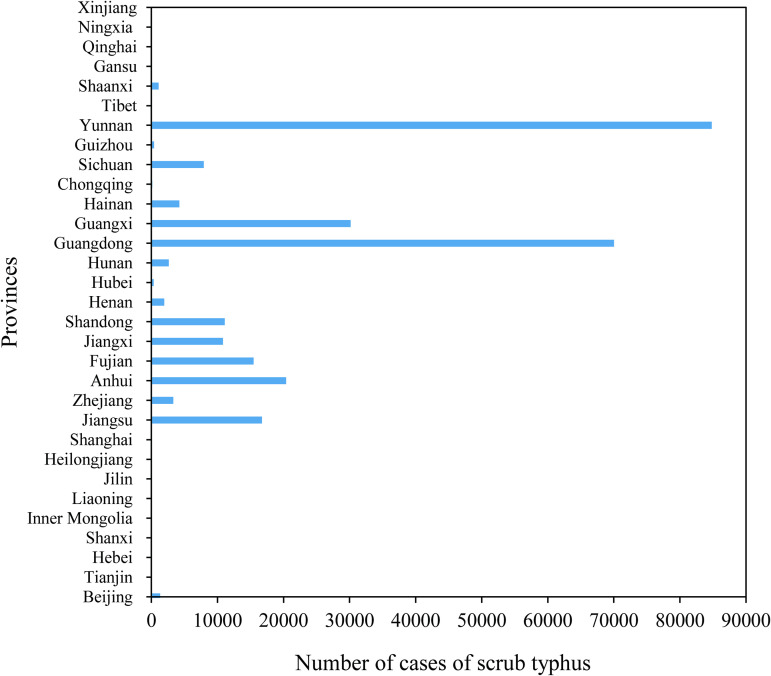
Reported cases of scrub typhus of 31 provinces in China.

In 2006, scrub typhus cases were reported from 226 counties (districts) across 16 provinces (autonomous regions) in China, totaling 1,244 cases. Only 32 counties (14%, 32/226) reported ≥10 cases, while 101 counties (45%, 101/226) documented single-case occurrences. By 2023, the disease had spread to 1,150 counties (districts) in 29 provinces (autonomous regions), with 33,400 reported cases. From 2006 to 2023, the top five provinces (municipalities) with the highest number of reported scrub typhus cases in China were Yunnan Province (84,795 cases), Guangdong Province (70,013 cases), Guangxi Zhuang Autonomous Region (30,147 cases), Anhui Province (20,398 cases), and Jiangsu Province (16,747 cases), collectively accounting for 78.40% of the national total cases (Fig 2).

### Time-series analyses for scrub typhus

**Fig 3:**
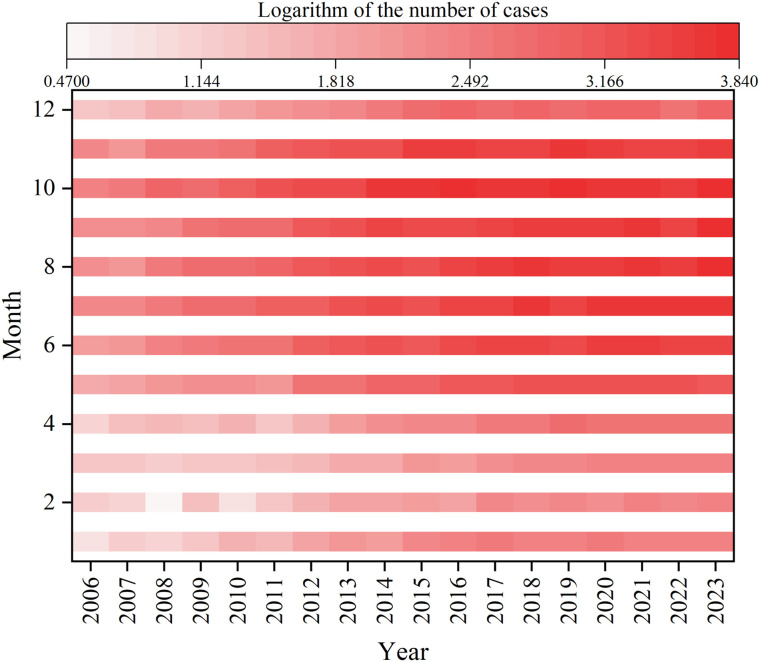
Heat map of monthly cases with scrub typhus in China from 2006 to 2023.

The annual case counts of scrub typhus in China from 2006 to 2023 demonstrated a statistically significant upward temporal trend. While infections occurred throughout the year, cases were predominantly concentrated between June and November, accounting for 88.45% of total reported incidence. October represented the epidemic peak with 59,670 cases, constituting 21.06% of cumulative reports (Figs 1 & 3).

**Fig 4:**
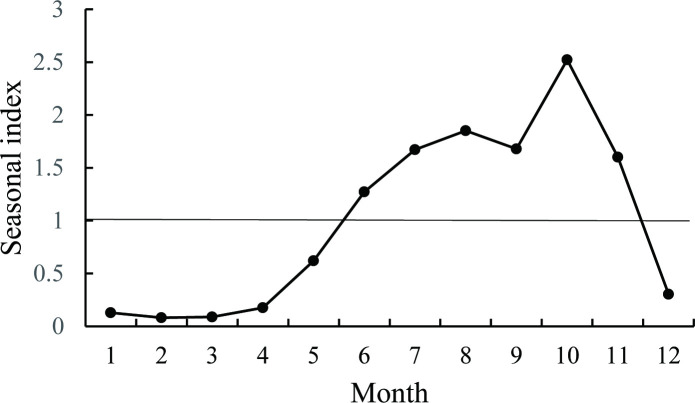
The seasonal index of scrub typhus in China from 2006 to 2023.

Analysis of monthly seasonal indices revealed distinct periodicity in scrub typhus epidemiology. The highest seasonal index (2.53) was observed in October, corresponding to a monthly average incidence of 0.033 per 100,000 population. Conversely, February displayed the lowest seasonal index (0.075) with minimal monthly incidence (0.001 per 100,000 population), as detailed in Fig 4.

### Spatial autocorrelation analyses for scrub typhus

**Fig 5:**
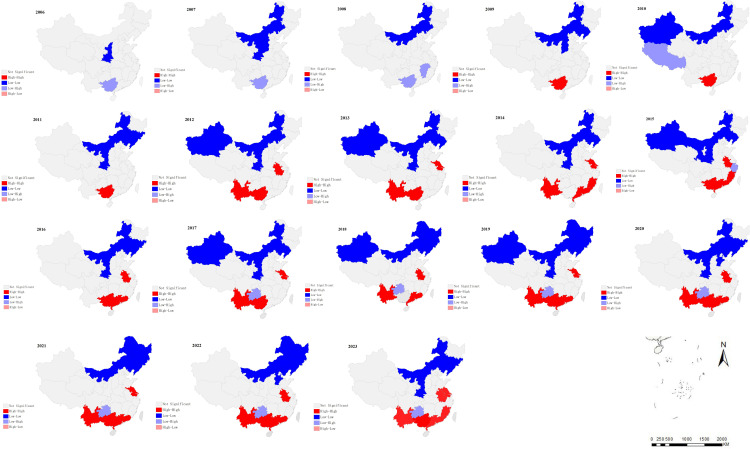
Local indicators of spatial association cluster map of local autocorrelation analysis on scrub typhus incidence in China, 2006 − 2023 (The map was created in ArcGIS software (version 10.8; ESRI Inc., Redlands, CA, USA; available at https://www.esri.com/), utilizing a public domain dataset from Natural Earth (https://www.naturalearthdata.com/) and adhering to the CC BY 4.0 license).

The results of global spatial autocorrelation analysis of scrub typhus incidence in China from 2006 to 2023 are shown in Table 2. The Moran’s *I* of each year was bigger than 0. Except for 2006–2011, the global spatial autocorrelation analysis of the scrub typhus incidence showed a significant global correlation (*P* < 0.05) (Table 2). The LISA cluster maps showed expanding “high-high” clusters in China’s southern regions, which were primarily centered there, including Yunnan, Guangdong, Guangxi, Anhui, Jiangsu and Fujian. While “low-low” clusters were growing in the northern areas, primarily in these regions, including Inner Mongolia, Xinjiang, Heilongjiang, Jilin, Liaoning and Qinghai (Fig 5).

**Table 2:** Spatial autocorrelation analysis of scrub typhus incidence in 31 provinces of mainland China (2006-2023).

Year	Moran’s *I*	Z-Score	*P*-value	Year	Moran’s *I*	Z-Score	*P*-value
2006	0.040	0.826	*P* > 0.05	2015	0.215	2.284	*P* < 0.05
2007	0.044	0.918	*P* > 0.05	2016	0.172	2.113	*P* < 0.05
2008	0.129	1.536	*P* > 0.05	2017	0.208	2.833	*P* < 0.05
2009	0.060	1.147	*P* > 0.05	2018	0.184	2.789	*P* < 0.05
2010	0.050	1.059	*P* > 0.05	2019	0.202	2.582	*P* < 0.05
2011	0.073	1.061	*P* > 0.05	2020	0.226	3.078	*P* < 0.05
2012	0.140	1.855	*P* < 0.05	2021	0.136	2.650	*P* < 0.05
2013	0.151	2.065	*P* < 0.05	2022	0.116	2.562	*P* < 0.05
2014	0.156	2.134	*P* < 0.05	2023	0.074	2.232	*P* < 0.05

### Spatiotemporal clustering analysis

**Fig 6:**
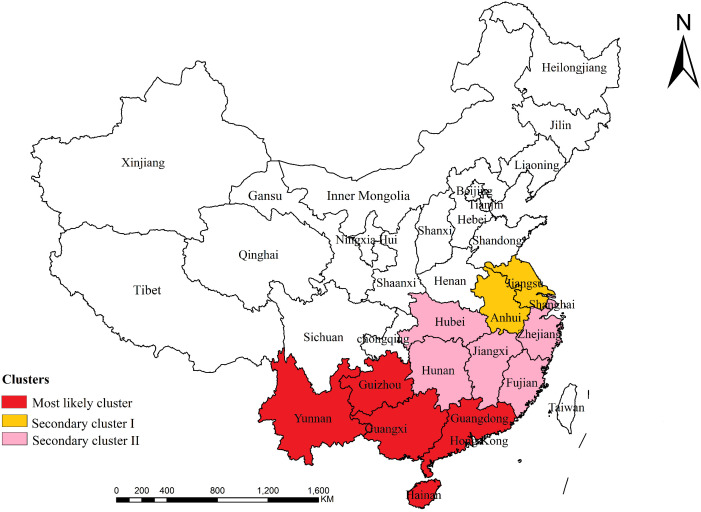
Space-time clusters of cases of scrub typhus at the provincial level in China from 2006 to 2023 (The map was created in ArcGIS software (version 10.8; ESRI Inc., Redlands, CA, USA; available at https://www.esri.com/), utilizing a public domain dataset from Natural Earth (https://www.naturalearthdata.com/) and adhering to the CC BY 4.0 license).

Using Kulldorff’s space-time scan statistic to detect spatiotemporal clusters across the study period. The most likely cluster was in the southern provinces in China, including Guangdong, Guangxi, Hainan, Guizhou, Yunnan. The time frame of the most likely cluster was between January 2015 and December 2023 and the relative risk (RR) of scrub typhus infection for people inside the cluster was 8.78 (LLR = 149974.86, p < 0.001). Besides, there were two significant secondary clusters were detected (Table 3). All clusters are indicated on the map in Fig 6.

**Table 3:** Analysis of spatiotemporal clustering of reported cases of scrub typhus in 31 provinces of mainland China (2006-2023).

Cluster type	Cluster time frame	Location/radius (km)	Observed cases	Expected cases	RR	LLR	*P* value
Most likely cluster	2015/1- 2023/12	Guangdong, Guangxi, Hainan, Guizhou, Yunnan/ 598.23	157563	35383.57	8.78	149974.86	<0.001
Secondary cluster I	2015/1-2020/12	Jiangsu, Anhui/ 155.56	21373	12887.25	1.86	3477.94	<0.001
Secondary cluster II	2007/1- 2009/12	Fujian, Jiangxi, Zhejiang, Hubei, Shanghai, Hunan/ 693.91	1105	916.55	3.19	1814.30	<0.001

RR = relative risk; LLR = log likelihood ratio.

### Association between scrub typhus incidence and environmental factors

The results of the negative binomial regression analysis, presented in Table 4, reveal significant associations between scrub typhus incidence and several potential influencing factors. The incidence rate ratio (IRR) for temperature with a 1-month lag was 1.17 (95% CI: 1.15, 1.19), with a highly significant p-value of less than 0.001. This indicates that for 1°C increase in temperature, the incidence of scrub typhus increased by 17%, after accounting for a 1-month lag. This strong positive association suggests that higher temperatures in the preceding month significantly contribute to the incidence of scrub typhus. Similarly, The IRR for rainfall with a 2-month lag was 1.008 (95% CI: 1.005, 1.010; p = 0.002). This implies that for 1mm increase in rainfall, the incidence of scrub typhus increased by 0.8%, after considering a 2-month lag. The significant p-value indicates that rainfall levels two months prior have an impact on the incidence of the disease. The IRR for NDVI was 1.07 (95% CI: 1.01, 1.10; p = 0.008). This suggests that for every unit increase in NDVI, the incidence of scrub typhus increased by 7%, indicating that areas with higher vegetation density may have a higher risk of the disease. The incidence of scrub typhus in adjacent provinces was a significant predictor of local incidence (IRR = 1.05, 95% CI: 1.03, 1.08; p = 0.013), which indicates that for every unit increase in the incidence of scrub typhus in adjacent provinces, the incidence in the study area increased by 5% (Table 4).

**Table 4:** The association between scrub typhus incidence and potential influencing factors by negative binomial regression.

Variable	IRR	95% CI	*P*-value
Temperature (1-month lag) (°C)	1.17	(1.15, 1.19)	<0.001
Rainfall (2-month lag) (mm)	1.008	(1.005, 1.010)	0.002
NDVI (Normalized Difference Vegetation Index)	1.07	(1.01, 1.10)	0.008
Adjacent province incidence	1.05	(1.03, 1.08)	0.013

### Analysis on the years of potential life lost of scrub typhus

Between 2006 and 2023, there were 103 deaths of residents in China. Of these deaths, 52 (50.49%) were of people aged between 50 and 69 years. The years of potential life lost (YPLL) due to scrub typhus across different age groups among males and females in China are detailed in Table 5, respectively. From 2006 to 2023, In males, the age groups that most contributed to the losses were 40–49 years and 0–4 years (28.32% and 27.99%, respectively), while in females, the highest frequencies of YPLL were observed between 50 and 59 years old (40.97%) and 60–69 years old (21.27%) (Table 2). Years of potential life lost (YPLL) between 2006 and 2023, scrub typhus accounted for a total of 2 119.29 person-years of life lost. After temporal normalization, the average annual YPLL was 117.74 person-years (95% CI 109.2 -126.3). Expressed relative to population size, the national YPLL rate was 0.0084 per 100 000 population.

**Table 5:** Number of deaths, potential years of life lost, proportion of potential years of life lost due to scrub typhus, according to sex and age group China, 2006-2023.

Age groups (year)	Males				Females			
	Deaths (*d*_*i*_)	remaining years (*a*_*i*_)	YPLL (*a*_*i*_*d*_*i*_)	YPLL %	Deaths (*d*_*i*_)	remaining years (ai)	YPLL (*a*_*i*_*d*_*i*_)	YPLL %
0 ~ 4	5	73.48	367.4	27.99	1	78.38	78.38	9.54
5 ~ 9	0	68.48	0	0.00	0	73.38	0	0.00
10 ~ 14	0	63.48	0	0.00	0	68.38	0	0.00
15 ~ 19	0	58.48	0	0.00	1	63.38	63.38	7.72
20 ~ 24	1	53.48	53.48	4.07	1	58.38	58.38	7.11
25 ~ 29	0	48.48	0	0.00	0	53.38	0	0.00
30 ~ 39	2	40.98	81.96	6.24	0	45.88	0	0.00
40 ~ 49	12	30.98	371.76	28.32	2	35.88	71.76	8.74
50 ~ 59	15	20.98	314.70	23.97	13	25.88	336.44	40.97
60 ~ 69	13	10.98	142.74	10.87	11	15.88	174.68	21.27
70 ~ 79	8	0.98	7.84	0.60	10	5.88	58.8	7.16
80 ~ 89	3	-9.02	-27.06	-2.06	5	-4.12	-20.6	-2.51
Total	59		1312.82	100	44		821.22	100

YPLL: years of potential life lost.

## Discussion

Consistent with earlier research showing a sharp rise in scrub typhus cases in China [31–33], our study shows a notable increase in scrub typhus incidence in China, with cases rising 23-fold between 2006 and 2023. Climate change, more human activity in endemic areas, and advancements in diagnostic technology (which allow for better case identification and reporting) could all be contributing factors to the observed increase in reported cases. Scrub typhus, a natural-focus disease, had received insufficient attention in newly recognized endemic areas, such as northern China, Africa, and South America, where it was not previously identified or monitored [34,35]. More people became aware of scrub typhus after the first confirmed cases were found in China. This led to improved diagnostic tests and increased surveillance, which in turn led to more cases being identified and reported. Furthermore, as urbanization, globalization, and climate change occur, rodents carrying infected mites may spread their ranges [33,35]. In line with research from Southeast Asia, the prevalence of cases among farmers (76.11%) highlights the dangers of occupational exposure while engaging in outdoor activities [36,37]. The exposure to pathogen-carrying chigger mites would rise as a result of agricultural operations [38–40]. For instance, farmers engage in harvesting activities in the fields from August to October, exposing them to larval mite bites during this time.

Scrub typhus exhibits distinct seasonal distribution, with cases predominantly concentrated between June and November (88.45% of total incidence). The incidence begins to rise in June and peaks in October. Endemic regions of scrub typhus can be classified into summer, autumn, and winter types based on seasonal patterns [31,41–43]. Among these, the winter type accounts for only sporadic cases, while the summer and autumn types represent the primary seasonal patterns in China. The summer type, characterized by a single incidence peak in July and August (e.g., Sichuan and Yunnan provinces), is observed in some southern provinces (e.g., Guangdong, Guangxi, and Fujian) with dual incidence peaks in July and October. In contrast, the autumn type demonstrates a singular peak in October, as seen in provinces such as Shandong, Jiangsu, and Anhui [9,44]. The seasonal index analysis in this study revealed dual incidence peaks in July- August and October, likely attributable to the overlapping seasonal peaks of the two endemic types (summer and autumn). The seasonal divergence in disease incidence between these two types is primarily driven by variations in the activity periods of their respective chigger mite vectors. The summer type is predominantly distributed in traditional endemic areas south of 31^°^ N latitude, where *Leptotrombidium deliense* serves as the primary vector. In contrast, the autumn type is mainly associated with *Leptotrombidium scutellare*. The seasonal epidemiology of scrub typhus in mainland China aligns with international findings. For instance, Japan reports bimodal incidence peaks in April- June and October-December [28], while South Korea exhibits a single peak in October- November [45]. These differences likely reflect distinct dominant mite species and their life cycles. In northern Honshu Island (Japan), *Leptotrombidium pallidum* is the principal vector [46], whereas *L. scutellare* drives high incidence in South Korea [47].

The substantial annual increase and marked geographic expansion of scrub typhus in China may be partially attributed to improvements in surveillance systems and heightened clinical awareness within healthcare systems. However, these factors cannot fully account for the rapid case surge or the pronounced northward and westward spread observed in recent decades. Emerging evidence links this trend to environmental drivers and global climate change, including precipitation, sunshine duration, temperature, cropland coverage, and relative humidity [13,44,48]. A study by Li et al. employing a negative binomial regression model based on Poisson distribution analyzed risk factors for scrub typhus in Guangzhou City from 2006 to 2012. Their findings demonstrated statistically significant impacts of temperature, atmospheric pressure, rainfall, and solar radiation on disease incidence. Specifically, a 1^°^C temperature increase correlated with a 14.98% rise in incidence, while a 100 Pa increase in atmospheric pressure reduced incidence by 8.03% [18]. Additionally, socioeconomic factors— such as travel history, high-frequency outdoor activities, and residential proximity to grasslands, vegetable fields, or ditches—were identified as significant risk factors [2,13,44].

China’s scrub typhus epidemiological pattern exhibits notable regional variation, with the five provinces with the highest case-reporting rates (Yunnan, Guangdong, Guangxi, Anhui, and Jiangsu) together accounting for 78.44% of the nation’s total cases. In line with the results of earlier research on the distribution of scrub typhus in East Asia, this concentration emphasizes the disease’s close ties to local ecological and socioeconomic factors [49]. The dominance of southern provinces (Yunnan, Guangdong, and Guangxi) aligns with historical patterns, as their subtropical climates—characterized by warm temperatures, high humidity, and abundant vegetation—create ideal habitats for chigger mites (*Leptotrombidium* spp.), the primary vectors, and their rodent hosts [9]. But the rise of Anhui and Jiangsu, in eastern and central China, as high-incidence regions highlight scrub typhus’s inland and northward spread, which is in line with recent findings of its geographic diffusion associated with climate change [50,51]. This change could be a result of the combined effects of land-use changes (such as increased agricultural production), climate change, and growing human encroachment into areas that are ecologically conducive to vector proliferation [52].

These results support previous research that connected temperature, precipitation, and humidity to the spread of scrub typhus [33]. For instance, the persistently high incidence in Yunnan and Guangdong may be driven by prolonged warm seasons and frequent rainfall, which sustain vector populations. Simultaneously, studies from similar endemic settings have shown that socioeconomic factors, including outdoor labor, agricultural practices, and rural-urban mobility, probably increase exposure risks in these areas [53]. Anhui and Jiangsu’s inclusion in the top five also points to changing environmental suitability, which may be facilitated by global climate change-induced changes in precipitation patterns and temperatures, allowing vector establishment in formerly non-endemic regions [50,51].

The strong positive association between temperature (1-month lag) and scrub typhus aligns with global evidence. Studies in Guangzhou, China, demonstrated a 14.98% (95% CI: 13.65–16.33%) increase in incidence per 1°C temperature rise, while research in Fujian reported an IRR of 1.18 for temperature at a 1-month lag [18,54]. Potential mechanisms include enhanced vector activity, where optimal temperatures accelerate the life cycle of *Leptotrombidium mites* and replication of *Orientia tsutsugamushi* pathogens [54], and increased human exposure due to warmer conditions promoting outdoor activities and elevating contact with endemic foci. Notably, studies in Vellore, India, observed suppressed transmission at temperatures >30°C, indicating that region-specific thermal thresholds exist and are influenced by local mite ecology [55]. Rainfall with a 2-month lag was positively associated with incidence, consistent with multiregional evidence [55]. Rainfall boosts soil moisture, facilitating mite egg hatching and rodent host population expansion [55]. The 7% higher incidence per NDVI unit likely operates through habitat suitability, as denser vegetation supports larger rodent populations, thereby amplifying mite - host contact. Temperature and rainfall modulate vegetation phenology (e.g., leaf-area duration), creating time-lagged effects on transmission. Shandong-based studies confirmed NDVI impacts peak after a 2–3-week lag, aligning with our results [56]. The 5% risk increase per unit rise in neighboring province incidence underscores spatial contagion via cross - border transmission, where rodent migration or human movement may spread pathogens across administrative boundaries [57]. It is important to note that these analyses are only our preliminary study. Limitations of the study include covariates may be subject to measurement error or may not fully represent actual ecological exposures (e.g., NDVI represents vegetation, but the specific habitats of chiggers may be more nuanced); Lagged effects were not fully considered (e.g., the impact of climate on incidence may be delayed by several months); And there may be unmeasured confounding factors.

Significant demographic differences in scrub typhus mortality are revealed by the YPLL study, highlighting the disease’s varying effects on age and gender groups. The concentration of YPLL burden and mortality (50.49%) among those aged 50–69 years is consistent with global trends of older populations experiencing more severe cases of scrub typhus, which are probably caused by immunosenescence and associated diseases. Notably, the YPLL patterns varied considerably by gender, with females showing peaks in the 50–59 and 60–69 age groups (40.97% and 21.27%, respectively), while males showed the highest losses in the 40–49 and 0–4 age groups (28.32% and 27.99%, respectively). These results imply that scrub typhus burden is disproportionately caused by premature mortality in younger males and older females, which are impacted by immunological senescence, occupational risks, and delayed diagnosis.

Particularly in southwestern China, where pediatric cases are more common, the raised YPLL in younger males (0–4 years) may be associated with immature immunity and increased exposure risks in endemic rural settings. Given that 76.11% of cases involved farmers, agricultural occupations (such as farming and forestry) probably enhance exposure to chigger mites among working-age males (40–49 years old) [52]. On the other hand, older females with YPLL have a longer life expectancy (80.88 years in 2023 compared to 75.98 years for males) and are more likely to experience serious consequences like shock and multiple organ dysfunction syndrome (MODS).

This study provides the first nationally representative estimates of premature mortality attributable to scrub typhus in mainland China. Although the absolute number of deaths was small, the average annual loss of 117.7 person-years underscores a persistent, if modest, burden over the 18-year period. When contextualised by population size, the national YPLL rate of 0.0084 per 100 000 population is markedly lower than the corresponding figure for dengue or tuberculosis [14], suggesting that scrub typhus currently contributes a minor share to China’s overall infectious-disease mortality. Nonetheless, the southern (e.g., Guangdong) and south-western (e.g.,Yunnan) provinces—where incidence are highest—highlight the need for targeted vector-control programmes and early clinical recognition to prevent further, potentially larger, losses in these endemic areas.

Some limitations of our study should be mentioned. In our study, the calculation of YPLL is based on a relatively small number of deaths (n = 103) over a long period (from 2006 to 2023), reflecting the low overall mortality of scrub typhus in China within the surveillance system. As highlighted in the epidemiological literature, YPLL is a valuable metric for quantifying the burden of premature mortality by incorporating both the number of deaths and the age at death, which traditional indicators like crude mortality rates cannot fully capture. However, its accuracy can be compromised when death data are sparse, as small sample sizes increase the risk of random variation distorting estimates, especially for infectious diseases with inherently low mortality rates [58]. Therefore, the YPLL analysis in this study may be subject to instability and potential bias, particularly in the context of low-fatality diseases like scrub typhus. Additionally, the comparisons of YPLL between sexes or age groups are preliminary and should not be overemphasized, as they are based on limited data points. Second, While China’s reported CFR is exceptionally low, this likely reflects nationwide improvements in early antibiotic therapy and diagnostics rather than systematic underreporting. Nevertheless, asymptomatic/mild fatal cases may be missed due to nonspecific presentations, warranting ongoing surveillance refinement.

## Supporting information

S1 Text The summary of the case definition. (DOCX)

S1 Data The underlying numerical data for Table 1. (XLSX)

S2 Data The underlying numerical data for Fig 1. (XLSX)

S3 Data The underlying numerical data for Fig 3. (XLSX)

S4 Data The underlying numerical data for Fig 4. (XLSX)

S5 Data The underlying numerical data of male for Table 5. (XLSX)

S6 Data The underlying numerical data of female for Table 5. (XLSX)
