# Supplementary material for: Epidemiologic features and potential year of life lost of scrub typhus in China: A nationwide surveillance analysis (2006– 2023)
Source: PLoS Negl Trop Dis. 2025 Oct 29;19(10):e0013666. doi: 10.1371/journal.pntd.0013666 (PMC12588475; doi:10.1371/journal.pntd.0013666)
Supplement: S1 Text — (DOCX) [file pntd.0013666.s001.docx]

The following are the case classification and diagnostic criteria.

**Case definition**

1 Epidemiological history: a person who has been in or visited a tsutsugamushi endemic area within 3 weeks prior to the onset of the disease and has a history of field activities.

2 Clinical manifestations:

2.1 A person had clinical symptoms of fever;

2.2 A person had lymphadenopathy;

2.3 A person had skin rash;

2.4 A person had specific eschar or ulcers.

3 Laboratory tests:

3.1 single serum OX-K agglutination titer ≥ 1:160 in the WeilFelix test;

3.2 A 4-fold or greater increase in serum IgG antibodies during the acute and recovery phases, as measured by indirect immunofluorescence antibody detection (IFA).

3.3 Positive results of O. tsutsugamushi by PCR;

3.4 Isolation of tsutsugamushi pathogens from clinical samples.

The diagnostic criteria for **suspected cases** meet 1, 2.1 plus 2.2 or 2.3.

The diagnostic criteria for **clinically confirmed cases** meet suspected cases plus 2.4; or both 1, 2.1, and 2.4.

The diagnostic criteria for **laboratory confirmed cases** meet suspected cases plus 3.2 or 3.3 or 3.4; or meet clinically confirmed cases plus any one of 3.
